# Associations between the choroid plexus and tau in Alzheimer’s disease using an active learning segmentation pipeline

**DOI:** 10.1186/s12987-024-00554-4

**Published:** 2024-07-12

**Authors:** Jiaxin Li, Yueqin Hu, Yunzhi Xu, Xue Feng, Craig H. Meyer, Weiying Dai, Li Zhao, for the Alzheimer’s Disease Neuroimaging Initiative

**Affiliations:** 1 College of Biomedical Engineering & Instrument Science, Zhejiang University, Hangzhou, Zhejiang China; 2 Psychology, Beijing Normal University, Beijing, China; 3 Biomedical Engineering, University of Virginia, Charlottesville, VA US; 4 Department of Computer Science, State University of New York at Binghamton, Binghamton, NY US

**Keywords:** Glymphatic system, Cerebrospinal fluid, Choroid plexus, Alzheimer’s disease, Active learning

## Abstract

**Background:**

The cerebrospinal fluid (CSF), primarily generated by the choroid plexus (ChP), is the major carrier of the glymphatic system. The alternations of CSF production and the ChP can be associated with the Alzheimer’s disease (AD). The present work investigated the roles of the ChP in the AD based on a proposed ChP image segmentation pipeline.

**Methods:**

A human-in-the-loop ChP image segmentation pipeline was implemented with intermediate and active learning datasets. The performance of the proposed pipeline was evaluated on manual contours by five radiologists, compared to the FreeSurfer and FastSurfer toolboxes. The ChP volume and blood flow were investigated among AD groups. The correlations between the ChP volume and AD CSF biomarkers including phosphorylated tau (p-tau), total tau (t-tau), amyloid-β42 (Aβ42), and amyloid-β40 (Aβ40) was investigated using three models (univariate, multiple variables, and stepwise regression) on two datasets with 806 and 320 subjects.

**Results:**

The proposed ChP segmentation pipeline achieved superior performance with a Dice coefficient of 0.620 on the test dataset, compared to the FreeSurfer (0.342) and FastSurfer (0.371). Significantly larger volumes (*p* < 0.001) and higher perfusion (*p* = 0.032) at the ChP were found in AD compared to CN groups. Significant correlations were found between the tau and the relative ChP volume (the ChP volume and ChP/parenchyma ratio) in each patient groups and in the univariate regression analysis (*p* < 0.001), the multiple regression model (*p* < 0.05 except for the t-tau in the LMCI), and in the step-wise regression model (*p* < 0.021). In addition, the correlation coefficients changed from − 0.32 to − 0.21 along with the AD progression in the multiple regression model. In contrast, the Aβ42 and Aβ40 shows consistent and significant associations with the lateral ventricle related measures in the step-wise regression model (*p* < 0.027).

**Conclusions:**

The proposed pipeline provided accurate ChP segmentation which revealed the associations between the ChP and tau level in the AD. The proposed pipeline is available on GitHub (https://github.com/princeleeee/ChP-Seg).

**Supplementary Information:**

The online version contains supplementary material available at 10.1186/s12987-024-00554-4.

## Introduction

The glymphatic system provides a drainage pathway for the clearance of metabolism waste in the brain, in which cerebrospinal fluid (CSF) serves as the critical carrier [1], fluxing the interstitial fluid through the Aquaporin-4 channels. The process is believed to help wash out the hallmarks of Alzheimer’s Disease (AD), including amyloid-β (Aβ) and tau protein, whose concentrations reflect pathological changes such as neuritic plaques and neuronal degeneration [2–4].

The choroid plexus (ChP) serves as the major secretory organ of CSF. Positioned on the floor of the ventricles, the ChP consists of fenestrated capillaries enveloped by a monolayer of epithelial cells. These epithelial cells are arranged in tight junctions, forming the blood-CSF barrier. Plasma from choroidal capillaries is transferred to the stroma and further filtered by the selective transporters of epithelial cells, finally flowing to ventricles as CSF [5]. CSF from the lateral ventricle (LVEN) successively passes through the third ventricle, and fourth ventricle to the subarachnoid space. CSF enters the brain parenchyma and participates in cleaning the metabolism waste through the glymphatic system.

Previous studies have also explored the potential associations between AD and ChP. The volume of the ChP was reported to be larger in AD patients compared to the control group (CN) [6, 7]. For example, Tadayon et al. reported the correlations between ChP volume and CSF biomarkers of amyloid-β42 (Aβ42), total tau (t-tau), and phosphorylated tau (p-tau) in AD patients [6]. In addition, Choi et al*.* reported the reduced permeability of the ChP in AD patients [7]. ChP enlargement is also observed in other aging-related diseases. For example, ChP volume is negatively correlated with α-syn in Parkinson’s disease [6] and enlarged ChP volume was also found in patients with early psychosis [8] and multiple sclerosis [9, 10].

Although ChP volumes in AD have been reported, the ChP segmentation and quantification model can be improved further. First, low accuracy in ChP segmentation has been reported in the FreeSurfer toolbox (Athinoula A. Martinos Center for Biomedical Imaging, Boston, US) [11], which was an atlas-based method and widely used in the above studies. Consequently, inaccurate ChP segmentation will introduce additional variance in the subsequent analyses. For example, it may be one of the possible reasons that no correlation was detected between the ChP and Aβ42 using the multivariate regression model [6]. Although enhanced ChP segmentation methods were proposed through the Gaussian Mixture Model, these methods relied on image intensity, making them susceptible to performance degradation when artifacts are present [12, 13]. Second, the overall clearance efficiency of the glymphatic system is associated with the brain volume directly. Possible impacts of the cerebral volume among individuals should be considered. In addition, the blood flow of the ChP is associated with the CSF generation [14]. However, the ChP perfusion in AD has not been investigated with accurate ChP segmentation. Third, although the alternation of the ChP was investigated through all AD groups, its changes at each AD progression stage is unknown.

A critical step of ChP analysis is to locate its region accurately. Deep learning algorithms have shown promising results in medical image analysis tasks, such as convolutional neural networks and active learning [15, 16]. Medical image segmentation requires pixel-wise annotation from experienced radiologists, which can be tedious and costly. For example, the ChP annotation by a radiologist requires 20 ± 5 min [13]. Recently, there has been an emergence of research focused on brain segmentation based on MRI. Opfer et al. developed a 3D convolutional neural network that enhanced the stability of thalamus segmentation across MRI scanners [17]. The hippocampus segmentation was proposed by Chen et al. with a multi-layer feature learning module [18]. Rau et al. obtained precise segmentation of putamen using deep neural patchwork [19]. Beyond these targeted subcortical structures, the field of whole-brain MRI segmentation is also advancing. Yu et al. introduced UNesT which leverages hierarchical transformer encoders to enhance segmentation accuracy [20]. Coupe et al. developed a coarse-to-fine segmentation strategy employing two ensembles of 125 3D U-Nets [21]. Huo et al. introduced SLANT-27, a model combining outputs from 27 distinct fully convolutional networks for final segmentation [22]. Despite the advancements and the intricate architecture of these models, they lack support for ChP segmentation. Because of the research interests of ChP and CSF circulation in neurodegeneration and other diseases, an ChP segmentation pipeline is highly demanded. Although deep learning ChP segmentation models were proposed, it is challenging to achieve sufficient accuracy with a small number of manually labeled images [23, 24]. Fortunately, the Human-in-the-loop strategy [25, 26], which includes humans in the process of model training to reduce the data amount, can improve the model performance effectively.

Therefore, in the present work, a ChP segmentation pipeline was developed using the human-in-the-loop method (the model is available on https://github.com/princeleeee/ChP-Seg). The proposed pipeline provided accurate ChP segmentation compared to the conventional method, and it enables the detection of the subtle correlations between the ChP volume and the protein levels in the AD subgroups.

## Method

### Datasets for the ChP segmentation pipeline developments

1. LVEN intermediate training dataset. A total of 35 T1-weighted MRI from the 2012 MICCAI Multi-Atlas Labeling Challenge dataset [27] were in-house annotated (L.Z. with 10 years’ experience in MR image processing).
2. ChP training dataset. 50 T1-weighted MRI from Alzheimer’s Disease Neuroimaging Initiative (ADNI) database (adni.loni.usc.edu). These images were in-house labeled (L.Z. with 10 years’ experience in MR image processing) to provide ground truth segmentation of the ChP.
3. ChP multi-rater test dataset. 20 T1-weighted MRI from ADNI. Each image was labeled by five experienced radiologists (average 3 years’ experience) and the final ground truth was decided by a majority vote. Meanwhile, the images were also labeled by five trainees (1–2 years’ experience in MR image processing) to provide naive labels compared to the proposed ChP segmentation pipeline and experienced radiologists.

### Datasets for investigating the ChP-related features in AD

1. Subset-Trio: to investigate the relationship between ChP volume and proteins including p-tau, t-tau, and Aβ42. 806 subjects were selected from ADNI, including 156 CN subjects, 95 subjects with significant memory concerns (SMC), 272 subjects with early-mild cognitive impairment (EMCI), 155 subjects with late-mild cognitive impairment (LMCI), and 128 subjects with AD. The inclusion criteria were the ones with complete p-tau, t-tau, and Aβ42 measurements in the baseline screening records.
2. Subset-Aβ40: This subset was extracted to examine the association between the ChP volume and the CSF biomarker amyloid-β40 (Aβ40), which was not investigated in previous works. It comprises subjects from the ADNI who have Aβ40 and available baseline records. A total of 320 subjects were selected, including 191 CN, 99 subjects with MCI, and 30 subjects with AD.
3. Subset-ASL: 145 subjects were selected, including 88 CN, 36 MCI, and 21 AD. The inclusion criteria were the availability of arterial spin labeling (ASL) images, T1-weighted MRI, and acquisitions using GE scanners.

The demographic information and MRI protocols are summarized in Table 1 and 2.

**Table 1:** Three subsets from the ADNI dataset

Datasets	Group	N	Age	Male	Aβ42 (pg/mL)	t-tau (pg/mL)	p-tau (pg/mL)	Aβ40 (pg/mL)
Subset-trio	CN	156	73.5 ± 6.4	0.49	1395.19 ± 670.08	237.31 ± 90.39	21.68 ± 9.12	–
	SMC	95	72.0 ± 5.4	0.41	1370.40 ± 612.48	239.29 ± 93.19	21.79 ± 9.57	
	EMCI	272	71.1 ± 7.3	0.56	1175.45 ± 584.38	255.51 ± 121.76	24.18 ± 13.72	
	LMCI	155	72.1 ± 7.4	0.54	935.32 ± 492.43	308.65 ± 136.14	30.21 ± 15.02	
	AD	128	74.4 ± 8.4	0.60	717.39 ± 447.22	376.34 ± 155.60	37.13 ± 16.20	
	Total	806	72.4 ± 7.3	0.53	1121.15 ± 614.90	279.42 ± 131.66	26.63 ± 14.29	
Subset-Aβ40	CN	191	70.4 ± 6.2	0.35	–	–	–	18785.03 ± 5250.26
	MCI	99	70.7 ± 7.3	0.54				17784.14 ± 5373.90
	AD	30	72.0 ± 8.9	0.67				16183.00 ± 4939.15
	Total	320	70.7 ± 6.9	0.44				18231.44 ± 5305.46
Subset-ASL	CN	88	74.8 ± 8.0	0.45	–	–	–	–
	MCI	36	72.6 ± 8.0	0.56				
	AD	21	76.1 ± 5.8	0.71				
	Total	145	74.4 ± 7.8	0.52				

“-” means this item is not available

**Table 2:**
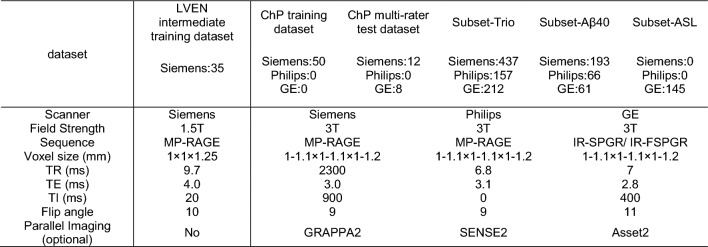
MRI protocols

### ChP segmentation pipeline

**Fig. 1:**
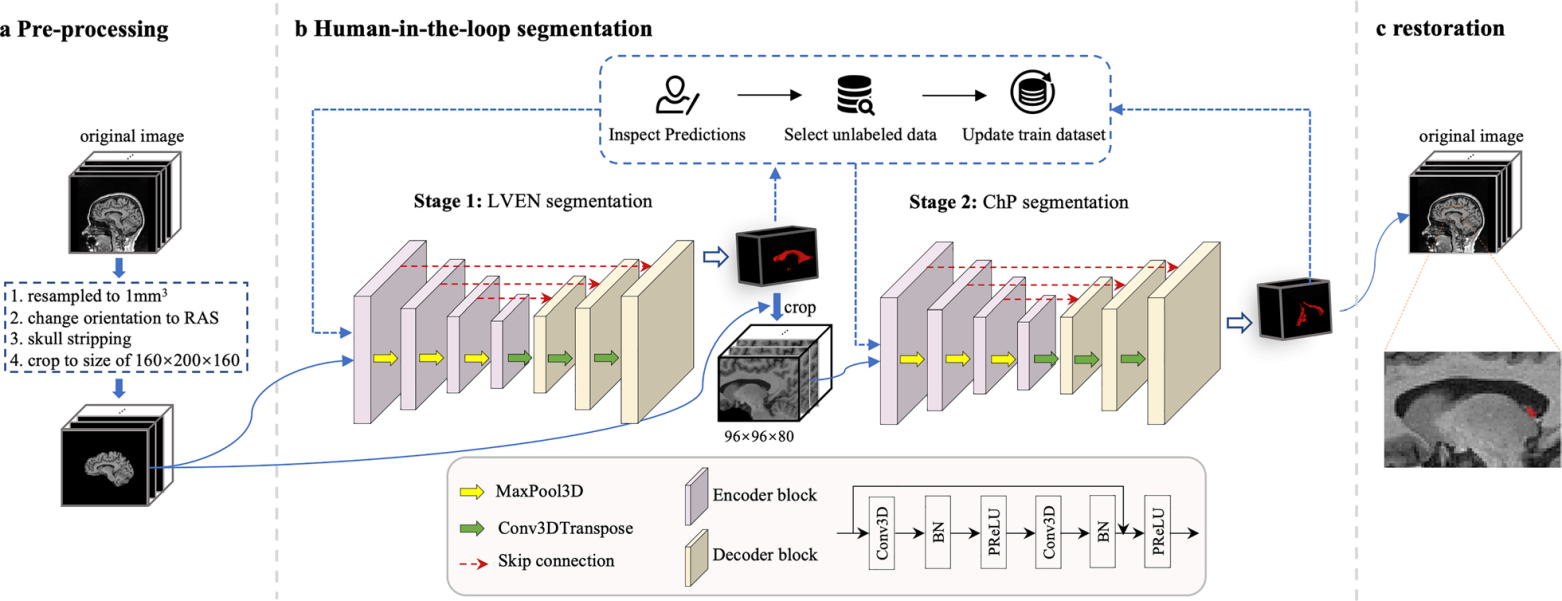
The proposed ChP segmentation pipeline. T1W MR image was pre-processed (**a**) and passed into the two-stage models with active learning correction (**b**). The ChP segmentation result was restored to the original image space (**c**)

The proposed segmentation pipeline consists of pre-processing, two-stage segmentation of LVEN and ChP, and restoration, Fig. 1. It was implemented in Keras (version 2.4.1).

In the pre-processing module, the original images were standardized chronologically. Firstly, the images were reoriented to the RAS (Right–Anterior–Superior) coordinate system and resampled to an isotropic resolution of 1 mm^3^. Skull stripping was performed using the Deepbrain [28] algorithm on T1-weighted MR images, resulting in a brain region mask by a threshold of 0.5. Subsequently, an image patch of 160 × 200 × 160 voxels was cropped based on the center of the brain mask. This cropping step aimed to balance the foreground and background by removing the background areas based on prior knowledge.

The two-stage segmentation model shared the same architecture, optimized 3D U-Net [29], which includes residual connections and the PReLU activation function (detailed model structures were provided in Table S1). The output of the LVEN segmentation was cropped to a 96 × 96 × 80 voxel patch, which served as the input of the ChP segmentation model. For both models, the dice loss was used to train the model in 200 epochs. The batch size was set to 2. The training process was initiated by a learning rate of 3 × 10^–4^, which was decreased by a factor of 0.5 when the loss function was on a plateau for 30 epochs. To avoid the overfitting of the models, data augmentation was performed by randomly flipping the images. Five-fold cross-validation was used to evaluate the stability of the model. With the selected hyperparameters, the model was retrained on the entire datasets of the LVEN intermediate train dataset and the ChP train dataset from scratch. 

Active learning strategy was adapted to improve the performance of models on the ADNI data. The predictions of the ADNI data inferred by intermediate models were inspected by the human expert (J.L. with 5 years’ experience in deep learning). Specifically, the segmentation of the LVEN and ChP was evaluated according to structural continuity in the 3D view, including a C-shaped structure for the LVEN and ribbon-like structures for the ChP. The top-15 worst segmentations were manually corrected and used to extend the current training dataset. This active learning procedure can be repeated until no obvious error is found by the human expert. In this work, 15 worst cases from Subset-Trio were selected, manually corrected, and then added to the training data to improve the model. It should be noted that the active learning operation was only required in the development stage of the models, and not needed in the deployment stage of the pipeline. 

The performance of the proposed pipeline, FreeSurfer (version 7.2.0), and its deep learning approach, FastSurfer (version 1.1.0) [30], were compared. The following metrics were employed to assess the efficacy of various methodologies, including the Dice coefficient, Jaccard coefficient, recall, and Hausdorff distance. The annotation consistency for intra-radiologist and intra-trainee was measured by the average Dice coefficients between each intra-pairwise combination on the ChP multi-rater test dataset.

### Statistical models

#### ChP and LVEN volumes in the AD

The Shapiro–Wilk test and Levene test were utilized to assess the normality and homogeneity of variance for LVEN and ChP volume in Subset-Trio, which was a prerequisite for further volumetric distribution comparison among the diagnostic groups (parametric method One-way ANOVA or non-parametric method Kruskal–Wallis followed by corresponding Post-Hoc multiple comparisons).

#### ChP perfusion functionality comparison

Gray matter (GM) was segmented from T1W using the SPM12 toolbox (Wellcome Trust Center for Neuroimaging, London, UK). Then the ASL was registered to the GM mask of the T1W and ChP was extracted using the mask from the proposed pipeline. The cerebral blood flow (CBF) of the brain was calculated using the following formula [31].

$$CBF= \frac{6000\cdot \lambda \cdot ASL\cdot {e}^{\frac{PLD}{{T}_{1,blood}}}}{2\cdot \alpha \cdot {T}_{1,blood}\cdot PD\cdot (1-{e}^{-\frac{\tau }{{T}_{1,blood}}})}$$

Here, $\lambda$ (mL/g) stands for brain blood partition coefficient, *ASL* is the signal from ASL scan, *PLD* is the time interval between labeling pulse ends and image acquisition starts in seconds, ${T}_{1,blood}$ is the longitudinal relaxation time of blood in the brain, $\alpha$ is the labeling efficiency (typically 0.95), *PD* is the signal in the proton density-weighted image, $\tau$ is label duration in seconds. Relative perfusion of ChP was calculated by dividing its CBF with the CBF of the GM region.

Normality and homogeneity of variance for the different groups within Subset-ASL were checked using the Shapiro–Wilk test and Levene test. Based on the results, the Mann–Whitney U or *t-test* will be used to compare the CBF between AD and CN.

#### Relationship between ChP volume and protein levels

To investigate the relationship between ChP volume and the levels of four proteins (p-tau, t-tau, Aβ42, and Aβ40), three models were developed. First, a linear regression model with the protein level as the dependent variable and ChP volume as the independent variable. Second, a multiple linear regression model including age, gender, LVEN volume, and brain parenchyma volume (computed by the FreeSurfer which excludes cerebellum, brain stem, CSF, ventricle, and ChP). Third, a stepwise regression model included ChP/parenchyma ratio, ChP/LVEN ratio, ChP volume, LVEN volume, age, gender, and parenchyma volume.

The statistical analysis was performed using SPSS 26.0 software.

## Results

### ChP segmentation accuracy

**Fig. 2:**
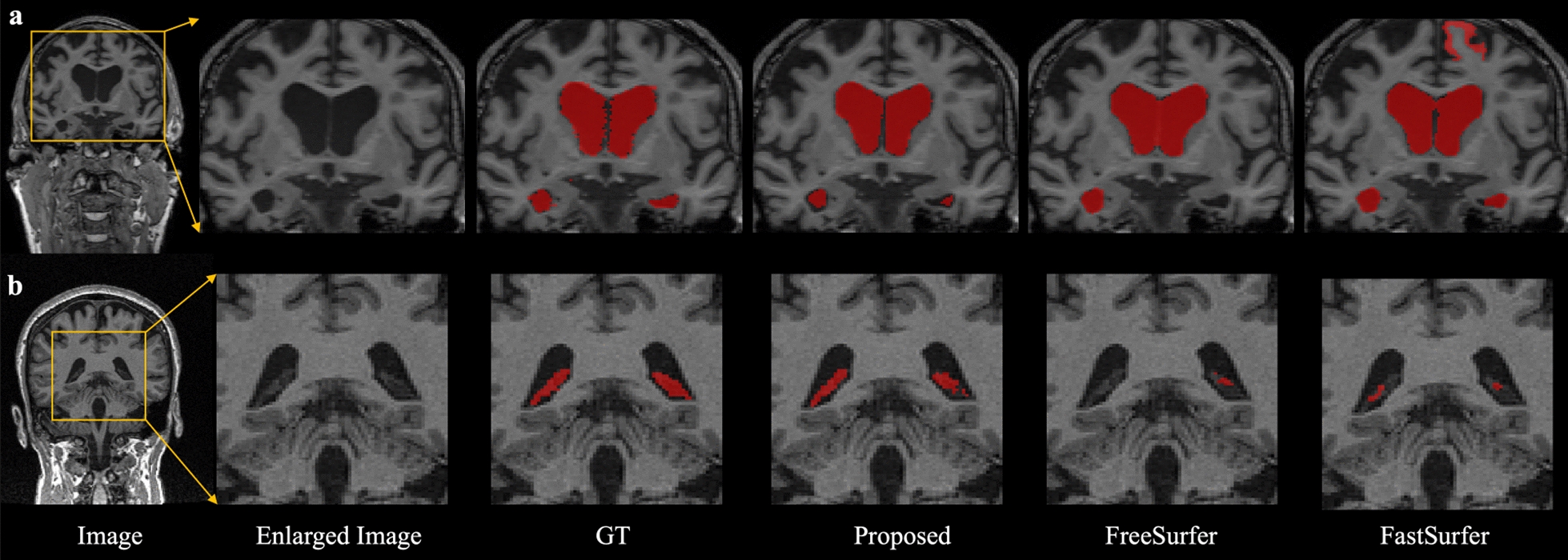
Segmentation results of the LVEN (**a**) and ChP (**b**) for selected subjects. The ground truth (GT) was votes from five radiologists

The mean dice of radiologists’ manual annotation was 0.884 and the consistency was 0.800 among the five radiologists. In contrast, the mean dice was 0.621 and the consistency was 0.608 among the five trainees. The whole human-in-the-loop was conducted twice. After the first iteration, extra 15 MRI from Subset-Trio were incorporated to enhance the models. The mean Dice coefficient of the proposed LVEN segmentation models was 0.825 in the five-fold cross-validation. In contrast, it was 0.805 in the FreeSurfer and 0.470 in the FastSurfer. The mean Dice coefficient of the proposed ChP segmentation models was 0.739 in the five-fold cross-validation. For ChP multi-rater test dataset, the proposed pipeline outperformed the FreeSurfer and the FastSurfer methods by 27.8% and 24.9% higher Dice coefficient, 24.8% and 22.6% higher Jaccard coefficient, 32.7% and 31.5% higher recall, and 66.3% and 53.5% lower Hausdorff distance, Table 3. The FreeSurfer underestimated the inferior horn of LVEN, while FastSurfer wrongly recognized some part of the gray matter as LVEN, Fig. 2a. Both the FreeSurfer and FastSurfer underestimated the ChP, Fig. 2b. In contrary, the proposed pipeline achieved more consistent segmentation as the overlapped regions of the radiologists.

**Table 3:** ChP segmentation performance quantitative comparison on the ChP multi-rater test dataset

Metrics	Dice coefficient	Jaccard coefficient	Recall	Hausdorff distance
Proposed	0.620	0.458	0.721	14.817
FreeSurfer	0.342	0.210	0.394	44.041
FastSurfer	0.371	0.232	0.406	31.859

### ChP volumes in AD

**Fig. 3:**
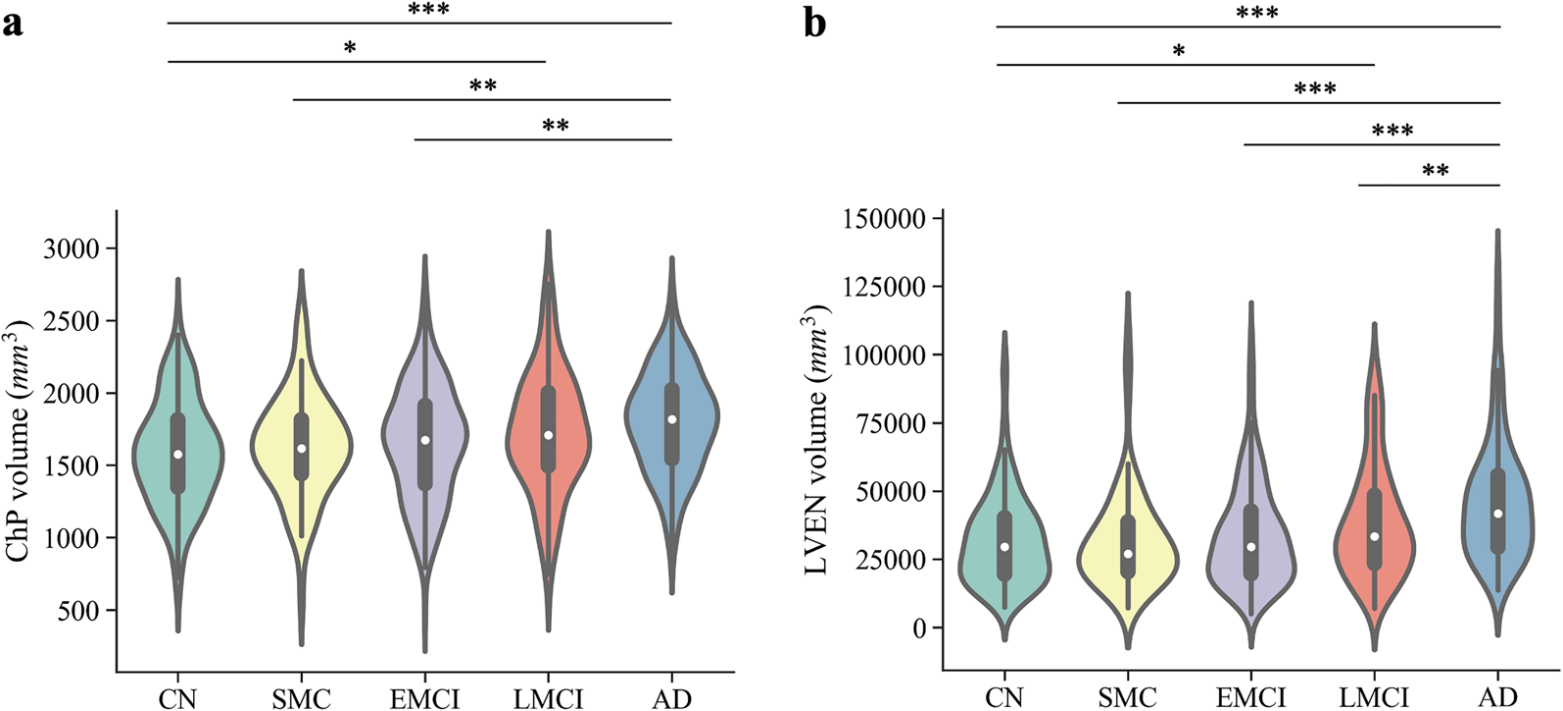
Volumetric distribution of ChP (**a**) and LVEN (**b**) in Subset-Trio. “*”, “**”, “***” stands for the p-values from 0.05 to 0.01, from 0.01 to 0.001, and less than 0.001 respectively

Figure 3 shows the distributions of ChP and LVEN volumes in each patient group. The Shapiro–Wilk test and Levene test showed that ChP volume in each group followed normal distributions and had homogeneous variances (*p* > 0.05, Table S2 and S3). One-way ANOVA indicated that the mean ChP volumes among the five groups were significantly different (*F* = 6.973, *p* < 0.001). Specifically, the ChP volumes in the AD and LMCI groups are significantly greater than in the CN group (Tukey Post Hoc Test, *p* < 0.05, Fig. 3a and Table S4).

The LVEN volume did not satisfy the normality and variance homogeneity tests (Tables S2 and S3). Non-parametric independent-samples Kruskal–Wallis test showed significant differences in LVEN volumes among the five groups (*H* = 53.351, *p* < 0.0001). Pairwise comparisons of groups revealed significant differences in the distribution of LVEN volume between SMC-AD, CN-LMCI, CN-AD, EMCI-AD, and LMCI-AD, Fig. 3b and Table S5.

### ChP blood flow in AD

**Fig. 4:**
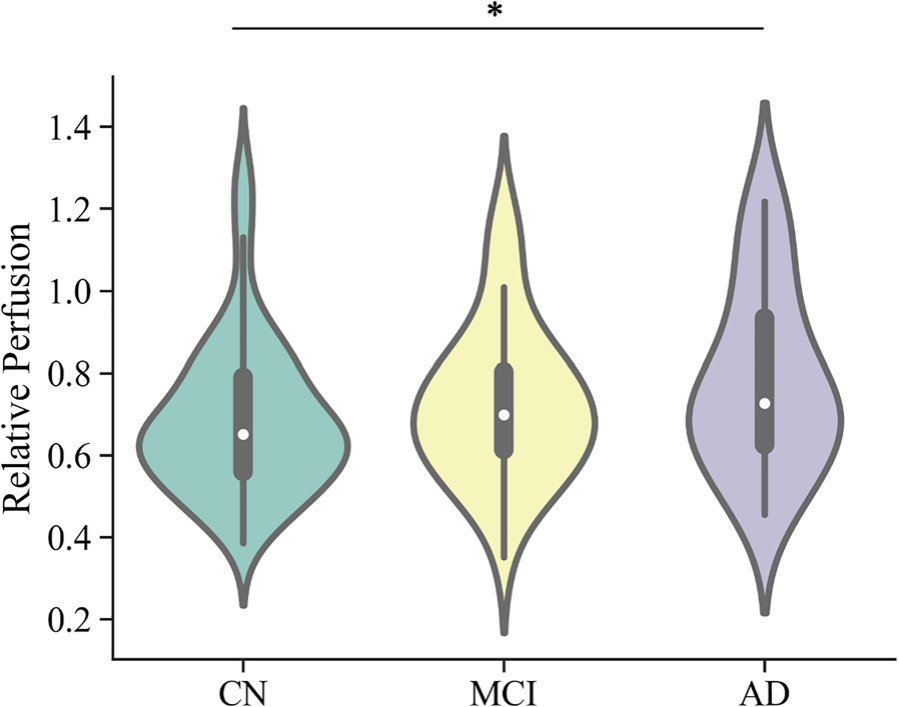
ChP relative perfusion distribution in Subset-ASL. “*” means the median of the two groups are different at the level of *p* < 0.05

The relative perfusion of ChP in the three groups showed homogeneous variance but the CN group did not follow normal distribution (refer to Table S6 and S7 in the supplementary material), Fig. 4. Non-parametric Mann–Whitney U-test showed that AD patients had significantly higher ChP relative perfusion compared to the CN group (*Z* = 682.0, *p* = 0.032).

### ChP volumes and protein levels

The histograms of protein levels showed normal distribution in Aβ40 and skew distributions in Aβ42, t-tau, and p-tau, Figure S1. Therefore, an arithmetic log transformation was applied to these proteins to meet the assumptions of the linear regression. Although significant correlations between the ChP volume and each protein level was found with all the subjects using the simple linear regression model, the associations may be not significant in each group, Table 4, Figure S2, and S3. Specifically, the ChP volume shows significant correlations with the p-tau and t-tau levels in each patient group, while it is not significantly correlated with the Aβ42 and Aβ40 in the CN, MCI, and AD groups.

**Table 4:** Simple linear regression between the ChP volume and p-tau, t-tau, Aβ42, and Aβ40

	p-tau		t-tau		Aβ42		Aβ40	
	β	*p*	β	*p*	β	*p*	β	*p*
All	− 0.136	**0.001**	− 0.151	**0.001**	− 0.263	**0.001**	− 0.196	**0.001**
CN	− 0.218	**0.006**	− 0.209	**0.009**	− 0.143	0.076	− 0.241	**0.001**
SMC	− 0.305	**0.003**	− 0.292	**0.004**	− 0.252	**0.014**		
EMCI	− 0.166	**0.006**	− 0.185	**0.002**	− 0.223	**0.001**	− 0.161	0.112
LMCI	− 0.216	**0.007**	− 0.226	**0.005**	− 0.273	**0.001**		
AD	− 0.359	**0.001**	− 0.376	**0.001**	− 0.156	0.078	0.028	0.882

β is the standardized coefficient in the regression model

^*^Bolded numbers indicated statistically significant *p*-values (*p* < 0.05)

Considering the LVEN volume, age, gender, and brain parenchyma volume (Pare), the ChP volume exhibited significant correlations with Aβ42, p-tau, and t-tau (*p* is from 0.012 to 0.001) in all subjects in the multiple linear regression model, Table 5. More importantly, the ChP volume is correlated with the p-tau and t-tau in most patient groups (*p* is from 0.027 to 0.001, except for the LMCI group) and the correlation coefficients, $\beta$, are generally reducing in their absolute values along with the AD progression. In contrast, the Aβ42 and Aβ40 levels are significantly correlated (*p* is from 0.017 to 0.001) with the LVEN volume mostly, except for the Aβ42 in the CN, EMCI and AD groups.

**Table 5:**
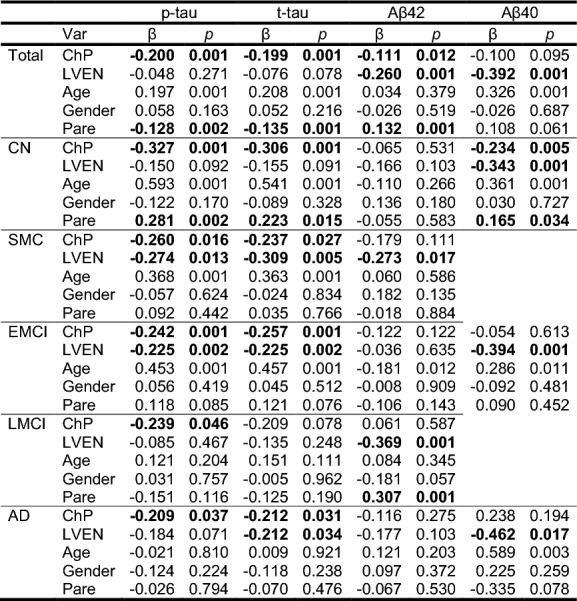
Multiple linear regression in each group on p-tau, t-tau, Aβ42, and Aβ40

β is the standardized coefficient in the regression model

^*^Bolded numbers indicated statistically significant variables along with their corresponding *p*-values

By controlling the colinear and excluding insignificant variables, the stepwise regression models show similar results as the multiple linear regression model with improved significance levels, Table 6. The ChP related variables, including ChP volume and ChP/parenchyma ratio, showed significant correlations (*p* is from 0.001 to 0.021) with p-tau and t-tau in each patient group. In contrast, the LVEN related variables show significant correlations (*p* is from 0.001 to 0.027) with the Aβ42 and Aβ40 levels, except for the Aβ42 in EMCI group. As expected, the age is also a significant contributor in most groups.

**Table 6:**
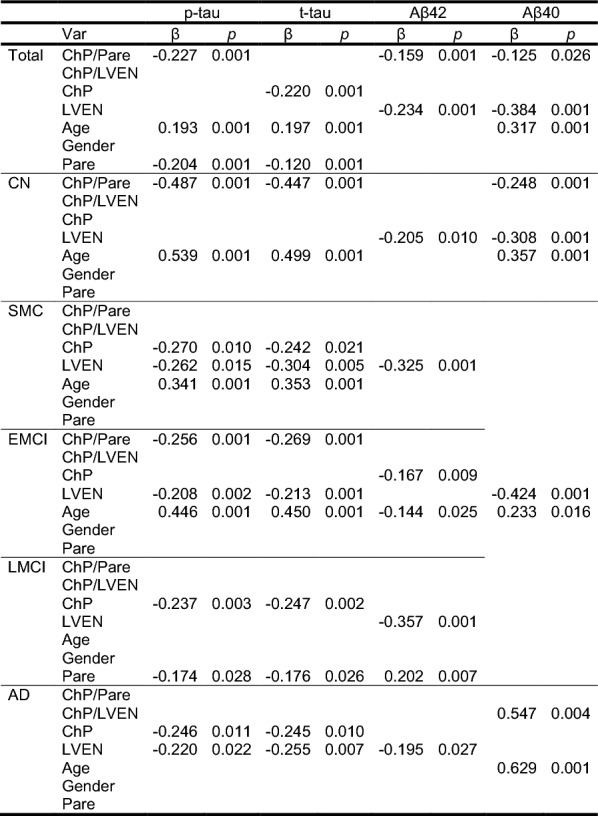
Stepwise regression in each group for p-tau, t-tau, Aβ42, and Aβ40

## Discussion

This study proposed a human-in-the-loop ChP segmentation pipeline, which improves the accuracy of ChP measures in AD studies. Based on the proposed pipeline, the work demonstrated sequentially increased ChP volume and perfusion with the progression of AD and significant correlations between the ChP related variables to p-tau and t-tau in entire and each patient groups.

The proposed human-in-the-loop ChP segmentation pipeline enabled accurate quantification of the relationship between ChP and AD. The proposed pipeline demonstrated a similar performance as the manual annotations provided by trainees, without inter-subject inconsistency. More importantly, it provided superior performances compared to the FreeSurfer and FastSurfer toolboxes. Due to the atlas-based segmentation approach, FreeSurfer may face challenges in segmenting enlarged and deformed ChP in AD patients. Since FastSurfer was trained under the supervision of FreeSurfer, it inherits similar pitfalls of the FreeSurfer methodology. In contrast, the proposed pipeline removed irrelevant anatomical regions using the two-step approach, which balanced the fore- and back-ground data. Because of the efficiency and success of 3D U-Net in medical image segmentation, it was selected as the foundational architecture for our pipeline. Meanwhile, the segmentation model was trained efficiently through a human intervention process. This intervention helps in minimizing the domain gap between train and test data in an efficient way. Based on the multi-rater labels, the proposed method outperformed the FreeSurfer and FastSurfer in the ChP segmentation and, therefore, it can reveal the subtle ChP changes related to AD protein biomarkers. In addition, the proposed pipeline is open-source online, which provides a ready-to-use pipeline for other ChP related studies.

Although LVEN segmentation was provided in the proposed pipeline, it can be replaced by other available methods. Since the ChP is a relatively small structure in the whole brain, the two-step segmentation strategy provided more balanced foreground and background in the ChP segmentation task. It can improve the accuracy of segmentation, as reported in the previous work [11]. Although the two-step segmentation was built as an all-in-one pipeline in the present work, the first step, LVEN segmentation, could be provided by established toolboxes. The LVEN segmentation mainly provides a coarse location of the ChP, which has a negligible impact on the ChP segmentation accuracy. However, the LVEN segmentation accuracy may directly affect the quantification of LVEN volume and statistical models with the protein levels of AD patients.

The enlarged ChP volume and increased blood flow in AD patients may reveal the compensation mechanism in the glymphatic system. The amount of CSF delivered to the brain parenchyma is closely associated with the cleaning of AD biomarkers. Our results demonstrate that ChP gets larger with the progression of AD, which is in line with the previous study [6]. Since the ChP volume was found to be related to the CSF production [32, 33], a larger ChP volume may indicate increased CSF secretion, which is used to clean the brain metabolism waste through the glymphatic system. This assumption can be further supported by the higher ChP perfusion in the AD. Eisma et al.’s work suggested that the ChP perfusion is positively correlated with the net CSF flow [14]. Although the total blood flow of the ChP was not measured directly in this work, the enlarged ChP volume and increased relative blood flow may suggest the increased total blood flow and therefore, the increased total CSF generation.

Establishing significant correlations between tau and ChP-related variables within each patient group may provide additional insights into the association between the ChP and AD. Although Tadayon et al.'s work [6] reported a correlation across all subjects, the variances explained by the ChP in each group was not explained thoroughly. The association at the group level can strengthen confirmation and provide additional insights. First, the increased ChP volume and abnormal tau level in AD patients were reported [6], which may indicate the compensation mechanism between the two. One possible explanation is that the tau protein could be trapped within the ChP capillaries, leading to the enlargement of the ChP as a compensatory mechanism to maintain its function [34]. This competitive interaction may be shown as a negative correlation between the ChP volume and the tau level in the CSF. Second, it is possible that the increased ChP volume indicates an improved clearance mechanism in the glymphatic system. Following this assumption, reduced correlations may suggest a breakdown in the cleansing system along with the AD progression. In other words, the enhanced clearance facilitated by the enlarged ChP volume may still be insufficient to effectively clear the excess tau proteins, resulting in increased tau levels in AD patients. However, decreased tau levels in AD patients were also reported in the longitudinal studies [35] [36]. Its interaction with the ChP requires further investigation.

Besides the absolute volume of ChP, the ratio of ChP volume and parenchyma volume may provide an effective measure of the clearance efficiency. The underlying complicated interaction among the absolute volumes of ChP, LVEN, and parenchyma in multiple regression models could potentially compromise the statistical analysis. The relative ChP volume, expressed as the ratio of ChP volume to both LVEN volume and parenchyma volume, can be used as an indicator of clearance efficiency. To avoid multicollinearity, a stepwise regression model was employed. The results showed significant correlations between the ratio of ChP and parenchyma volume and the AD protein biomarkers. This supports the hypothesis that an enlarged ChP volume and enhanced ChP perfusion may contribute to increased CSF production in the glymphatic system.

In the statistical models, the ChP-related variables exhibited a negative correlation with AD CSF biomarkers. These findings align with previous studies that reported a negative association between ChP volume and t-tau and p-tau levels [6]. This observation may suggest a correlation between heightened CSF production and the potential for improved metabolic clearance. Moreover, correlation between Aβ42 and Aβ40 and ChP volume in the multiple regression model has not been previously reported. This discrepancy might be attributed to the advantages offered by our proposed pipeline.

There are a few limitations in our study. First, the proposed pipeline primarily focused on the ChP located within the LVEN. Although the ChP in the third and fourth ventricles were not included due to the low physical image resolution, they may play an important role in CSF generation. In addition, CSF secretion may also happen at the ventricular ependyma and the blood–brain barrier [37, 38]. Therefore, this work may only illustrate the relationship between a portion of CSF generation and AD. Second, this work mainly investigated the space occupancy of the ChP rather than its actual volume. The ChP may appear folded within the ventricle because of limitations in MRI resolution. Therefore, further models are required to investigate the contributions of ChP volume or surface area to AD. Third, the present work did not integrate ChP perfusion and volume into the same statistic model due to the limited sample size. The contributions of both the enlarged ChP volume and increased ChP perfusion require further investigation. Forth, additional confounding variables may require further investigation. The Apolipoprotein E e4 genotype is a known contributor to the AD and may also be associated with ChP changes. Other behavioral factors, such as chronic drinking, active smoking, and sleep, might also influence AD progression [39, 40]. It has been reported that the glymphatic system becomes more active during sleep than when awake [41]. Consequently, sleep quality could impact the efficiency of metabolic clearance during sleep. In addition, the present study mainly focuses on the AD. Recent studies have revealed potential associations between ChP and other neurodegenerative diseases such as Parkinson’s disease and multiple sclerosis, which may benefit from the proposed ChP segmentation pipeline.

## Conclusion

Utilizing the proposed accurate ChP segmentation pipeline, we identified increased ChP volume and elevated ChP blood flow in AD. Noteworthy correlations between ChP-related biomarkers and tau protein were identified throughout the progression of AD. These findings highlight the important roles of ChP in the context of AD.

### Supplementary Information

Supplementary materials 1. Table S1. Model summary. Table S2. Leven tests for the homogeneity of variance on ChP and LVEN volume. Table S3: Normality tests of ChP and LVEN volume by Shapiro-Wilk test. Table S4. Tukey Post Hoc for pairwise means comparisons for ChP volumes after ANOVA test. Table S5. Post Hoc multiple comparisons of LVEN volume after non-parametric independent-samples Kruskal-Wallis test is significant. Table S6. Leven tests for the homogeneity of variance on relative perfusion. Table S7: Normality test of relative perfusion by Shapiro-Wilk test. Figure S1. Histogram of proteins level for Aβ42, Aβ40, t-tau, and p-tau. Figure S2. The fitted linear relationship between the protein level and ChP volume for each study group. β is the standardized regression coefficient. Figure S3. The fitted linear relationship between the protein level and ChP volume. β is the standardized regression coefficient.

## Data Availability

The present model and processed data will be provided online at https://github.com/princeleeee/ChP-Seg. The raw data are owned by the ADNI group.
