# Supplementary material for: Associations between the choroid plexus and tau in Alzheimer’s disease using an active learning segmentation pipeline
Source: Fluids Barriers CNS. 2024 Jul 12;21:56. doi: 10.1186/s12987-024-00554-4 (PMC11245807; doi:10.1186/s12987-024-00554-4)
Supplement: Supplementary file 1 — Supplementary materials 1. Table S1. Model summary. Table S2. Leven tests for the homogeneity of variance on ChP and LVEN volume. Table S3: Normality tests of ChP and LVEN volume by Shapiro-Wilk test. Table S4. Tukey Post Hoc for pairwise means comparisons for ChP volumes after ANOVA test. Table S5. Post Hoc multiple comparisons of LVEN volume after non-parametric independent-samples Kruskal-Wallis test is significant. Table S6. Leven tests for the homogeneity of variance on relative perfusion. Table S7: Normality test of relative perfusion by Shapiro-Wilk test. Figure S1. Histogram of proteins level for Aβ42, Aβ40, t-tau, and p-tau. Figure S2. The fitted linear relationship between the protein level and ChP volume for each study group. β is the standardized regression coefficient. Figure S3. The fitted linear relationship between the protein level and ChP volume. β is the standardized regression coefficient. [file 12987_2024_554_MOESM1_ESM.docx]

**Supplementary materials**

Table S1. Model summary.

| Layer (type) | Output Shape | Param # | Connected to | |
| --- | --- | --- | --- | --- |
| input_1 (InputLayer) | [(None, 160, 200, 160, 1)] | 0 |  |  |
| conv3d (Conv3D) | (None, 160, 200, 160, 8) | 224 | input_1[0][0] |  |
| layer_normalization () | (None, 160, 200, 160, 8) | 16 | conv3d[0][0] |  |
| p_re_lu (PReLU) | (None, 160, 200, 160, 8) | 40960000 | layer_normalization[0][0] |  |
| conv3d_1 (Conv3D) | (None, 160, 200, 160, 8) | 1736 | p_re_lu[0][0] |  |
| layer_normalization_1 () | (None, 160, 200, 160, 8) | 16 | conv3d_1[0][0] |  |
| concatenate (Concatenate) | (None, 160, 200, 160, 9) | 0 | layer_normalization_1[0][0] |  |
| p_re_lu_1 (PReLU) | (None, 160, 200, 160, 9) | 46080000 | concatenate[0][0] |  |
| max_pooling3d (MaxPooling3D) | (None, 80, 100, 80, 9) | 0 | p_re_lu_1[0][0] |  |
| conv3d_2 (Conv3D) | (None, 80, 100, 80, 16) | 3904 | max_pooling3d[0][0] |  |
| layer_normalization_2 () | (None, 80, 100, 80, 16) | 32 | conv3d_2[0][0] |  |
| p_re_lu_2 (PReLU) | (None, 80, 100, 80, 16) | 10240000 | layer_normalization_2[0][0] |  |
| conv3d_3 (Conv3D) | (None, 80, 100, 80, 16) | 6928 | p_re_lu_2[0][0] |  |
| layer_normalization_3 () | (None, 80, 100, 80, 16) | 32 | conv3d_3[0][0] |  |
| concatenate_1 (Concatenate) | (None, 80, 100, 80, 25) | 0 | layer_normalization_3[0][0] |  |
| p_re_lu_3 (PReLU) | (None, 80, 100, 80, 25) | 16000000 | concatenate_1[0][0] |  |
| max_pooling3d_1 (MaxPooling3D) | (None, 40, 50, 40, 25) | 0 | p_re_lu_3[0][0] |  |
| conv3d_4 (Conv3D) | (None, 40, 50, 40, 32) | 21632 | max_pooling3d_1[0][0] |  |
| layer_normalization_4 () | (None, 40, 50, 40, 32) | 64 | conv3d_4[0][0] |  |
| p_re_lu_4 (PReLU) | (None, 40, 50, 40, 32) | 2560000 | layer_normalization_4[0][0] |  |
| conv3d_5 (Conv3D) | (None, 40, 50, 40, 32) | 27680 | p_re_lu_4[0][0] |  |
| layer_normalization_5 () | (None, 40, 50, 40, 32) | 64 | conv3d_5[0][0] |  |
| concatenate_2 (Concatenate) | (None, 40, 50, 40, 57) | 0 | layer_normalization_5[0][0] |  |
| p_re_lu_5 (PReLU) | (None, 40, 50, 40, 57) | 4560000 | concatenate_2[0][0] |  |
| max_pooling3d_2 (MaxPooling3D) | (None, 20, 25, 20, 57) | 0 | p_re_lu_5[0][0] |  |
| conv3d_6 (Conv3D) | (None, 20, 25, 20, 64) | 98560 | max_pooling3d_2[0][0] |  |
| layer_normalization_6 () | (None, 20, 25, 20, 64) | 128 | conv3d_6[0][0] |  |
| p_re_lu_6 (PReLU) | (None, 20, 25, 20, 64) | 640000 | layer_normalization_6[0][0] |  |
| conv3d_7 (Conv3D) | (None, 20, 25, 20, 64) | 110656 | p_re_lu_6[0][0] |  |
| layer_normalization_7 () | (None, 20, 25, 20, 64) | 128 | conv3d_7[0][0] |  |
| concatenate_3 (Concatenate) | (None, 20, 25, 20, 121) | 0 | layer_normalization_7[0][0] |  |
| p_re_lu_7 (PReLU) | (None, 20, 25, 20, 121) | 1210000 | concatenate_3[0][0] |  |
| conv3d_transpose (Conv3DTranspose) | (None, 40, 50, 40, 32) | 31008 | p_re_lu_7[0][0] |  |
| concatenate_4 (Concatenate) | (None, 40, 50, 40, 89) | 0 | conv3d_transpose[0][0] |  |
| conv3d_8 (Conv3D) | (None, 40, 50, 40, 32) | 76928 | concatenate_4[0][0] |  |
| layer_normalization_8 () | (None, 40, 50, 40, 32) | 64 | conv3d_8[0][0] |  |
| p_re_lu_8 (PReLU) | (None, 40, 50, 40, 32) | 2560000 | layer_normalization_8[0][0] |  |
| conv3d_9 (Conv3D) | (None, 40, 50, 40, 32) | 27680 | p_re_lu_8[0][0] |  |
| layer_normalization_9 () | (None, 40, 50, 40, 32) | 64 | conv3d_9[0][0] |  |
| concatenate_5 (Concatenate) | (None, 40, 50, 40, 121) | 0 | layer_normalization_9[0][0] | |
| p_re_lu_9 (PReLU) | (None, 40, 50, 40, 121) | 9680000 | concatenate_5[0][0] | |
| conv3d_transpose_1 (Conv3DTranspose) | (None, 80, 100, 80, 16) | 15504 | p_re_lu_9[0][0] | |
| concatenate_6 (Concatenate) | (None, 80, 100, 80, 41) | 0 | conv3d_transpose_1[0][0] | |
| conv3d_10 (Conv3D) | (None, 80, 100, 80, 16) | 17728 | concatenate_6[0][0] | |
| layer_normalization_10 () | (None, 80, 100, 80, 16) | 32 | conv3d_10[0][0] | |
| p_re_lu_10 (PReLU) | (None, 80, 100, 80, 16) | 10240000 | layer_normalization_10[0][0] | |
| conv3d_11 (Conv3D) | (None, 80, 100, 80, 16) | 6928 | p_re_lu_10[0][0] | |
| layer_normalization_11 () | (None, 80, 100, 80, 16) | 32 | conv3d_11[0][0] | |
| concatenate_7 (Concatenate) | (None, 80, 100, 80, 57) | 0 | layer_normalization_11[0][0] | |
| p_re_lu_11 (PReLU) | (None, 80, 100, 80, 57) | 36480000 | concatenate_7[0][0] | |
| conv3d_transpose_2 (Conv3DTranspose) | (None, 160, 200, 160, 8) | 3656 | p_re_lu_11[0][0] | |
| concatenate_8 (Concatenate) | (None, 160, 200, 160, 17) | 0 | conv3d_transpose_2[0][0] | |
| conv3d_12 (Conv3D) | (None, 160, 200, 160, 8) | 3680 | concatenate_8[0][0] | |
| layer_normalization_12 () | (None, 160, 200, 160, 8) | 16 | conv3d_12[0][0] | |
| p_re_lu_12 (PReLU) | (None, 160, 200, 160, 8) | 40960000 | layer_normalization_12[0][0] | |
| conv3d_13 (Conv3D) | (None, 160, 200, 160, 8) | 1736 | p_re_lu_12[0][0] | |
| layer_normalization_13 () | (None, 160, 200, 160, 8) | 16 | conv3d_13[0][0] | |
| concatenate_9 (Concatenate) | (None, 160, 200, 160, 25) | 0 | layer_normalization_13[0][0] | |
| p_re_lu_13 (PReLU) | (None, 160, 200, 160, 25) | 128000000 | concatenate_9[0][0] | |
| conv3d_14 (Conv3D) | (None, 160, 200, 160, 1) | 26 | p_re_lu_13[0][0] | |
| predictions (Activation) | (None, 160, 200, 160, 1) | 0 | conv3d_14[0][0] | |
| Total params: 350,626,898 |  |  |  | |
| Trainable params: 350,626,898 |  |  |  | |
| Non-trainable params: 0 |  |  |  | |

Table S2. Leven tests for the homogeneity of variance on ChP and LVEN volume.

|  | ChP volume | | | |  | LVEN volume | | | |
| --- | --- | --- | --- | --- | --- | --- | --- | --- | --- |
|  | Levene | df1 | df2 | Sig. |  | Levene | df1 | df2 | Sig. |
| Mean | 1.562 | 4 | 801 | .183 |  | 3.125 | 4 | 801 | .015 |
| Median | 1.535 | 4 | 801 | .190 |  | 2.405 | 4 | 801 | .048 |
| Median & Adj df | 1.535 | 4 | 783 | .190 |  | 2.405 | 4 | 767 | .048 |
| trimmed mean | 1.562 | 4 | 801 | .182 |  | 2.922 | 4 | 801 | .020 |

Table S3: Normality tests of ChP and LVEN volume by Shapiro-Wilk test.

| Group | ChP volume | | |  | LVEN volume | | |
| --- | --- | --- | --- | --- | --- | --- | --- |
|  | Statistic | df | Sig. |  | Statistic | df | Sig. |
| CN | .993 | 156 | .634 |  | .911 | 156 | .000 |
| SMC | .988 | 95 | .513 |  | .829 | 95 | .000 |
| EMCI | .994 | 272 | .410 |  | .914 | 272 | .000 |
| LMCI | .992 | 155 | .571 |  | .922 | 155 | .000 |
| AD | .995 | 128 | .906 |  | .910 | 128 | .000 |

Table S4. Tukey Post Hoc for pairwise means comparisons for ChP volumes after ANOVA test.

| Group 1 | Group 2 | Mean Difference (I-J) | Std. Error | Sig. | 95% Confidence Interval | |
| --- | --- | --- | --- | --- | --- | --- |
|  |  |  |  |  | Lower Bound | Upper Bound |
| CN | SMC | -45.37989 | 49.36495 | .889 | -180.3437 | 89.5839 |
|  | EMCI | -68.58984 | 38.09615 | .374 | -172.7448 | 35.5651 |
|  | LMCI | -140.48346^*^ | 43.01877 | **.010** | -258.0968 | -22.8701 |
|  | AD | -216.26723^*^ | 45.23742 | **.000** | -339.9464 | -92.5881 |
| SMC | CN | 45.37989 | 49.36495 | .889 | -89.5839 | 180.3437 |
|  | EMCI | -23.20995 | 45.20565 | .986 | -146.8023 | 100.3824 |
|  | LMCI | -95.10357 | 49.42519 | .305 | -230.2321 | 40.0250 |
|  | AD | -170.88734^*^ | 51.36788 | **.008** | -311.3272 | -30.4475 |
| EMCI | CN | 68.58984 | 38.09615 | .374 | -35.5651 | 172.7448 |
|  | SMC | 23.20995 | 45.20565 | .986 | -100.3824 | 146.8023 |
|  | LMCI | -71.89362 | 38.17417 | .327 | -176.2618 | 32.4746 |
|  | AD | -147.67739^*^ | 40.65805 | **.003** | -258.8365 | -36.5182 |
| LMCI | CN | 140.48346^*^ | 43.01877 | **.010** | 22.8701 | 258.0968 |
|  | SMC | 95.10357 | 49.42519 | .305 | -40.0250 | 230.2321 |
|  | EMCI | 71.89362 | 38.17417 | .327 | -32.4746 | 176.2618 |
|  | AD | -75.78377 | 45.30315 | .451 | -199.6426 | 48.0751 |
| AD | CN | 216.26723^*^ | 45.23742 | **.000** | 92.5881 | 339.9464 |
|  | SMC | 170.88734^*^ | 51.36788 | **.008** | 30.4475 | 311.3272 |
|  | EMCI | 147.67739^*^ | 40.65805 | **.003** | 36.5182 | 258.8365 |
|  | LMCI | 75.78377 | 45.30315 | .451 | -48.0751 | 199.6426 |

* The mean difference is significant at the 0.05 level.

Table S5. Post Hoc multiple comparisons of LVEN volume after non-parametric independent-samples Kruskal-Wallis test is significant.

| Group 1- Group 2 | Test Statistic | Std. Error | Std. Test Statistic | Sig. | Adj. Sig. |
| --- | --- | --- | --- | --- | --- |
| SMC-CN | 8.502 | 30.299 | .281 | .779 | 1.000 |
| SMC-EMCI | -25.715 | 27.746 | -.927 | .354 | 1.000 |
| SMC-LMCI | -83.783 | 30.336 | -2.762 | .006 | .057 |
| SMC-AD | -177.016 | 31.528 | -5.615 | .000 | **.000** |
| CN-EMCI | -17.212 | 23.382 | -.736 | .462 | 1.000 |
| CN-LMCI | -75.280 | 26.404 | -2.851 | .004 | **.044** |
| CN-AD | -168.514 | 27.766 | -6.069 | .000 | **.000** |
| EMCI-LMCI | -58.068 | 23.430 | -2.478 | .013 | .132 |
| EMCI-AD | -151.301 | 24.955 | -6.063 | .000 | **.000** |
| LMCI-AD | -93.233 | 27.806 | -3.353 | .001 | **.008** |

Table S6. Leven tests for the homogeneity of variance on relative perfusion.

|  | Levene Statistic | df1 | df2 | Sig. |
| --- | --- | --- | --- | --- |
| Mean | .860 | 2 | 142 | .425 |
| Median | .498 | 2 | 142 | .609 |
| Median & Adj df | .498 | 2 | 139.540 | .609 |
| Trimmed mean | .878 | 2 | 142 | .418 |

Table S7: Normality test of relative perfusion by Shapiro-Wilk test.

| Group | Statistic | df | Sig. |
| --- | --- | --- | --- |
| CN | .911 | 88 | .000 |
| MCI | .963 | 36 | .264 |
| AD | .931 | 21 | .142 |


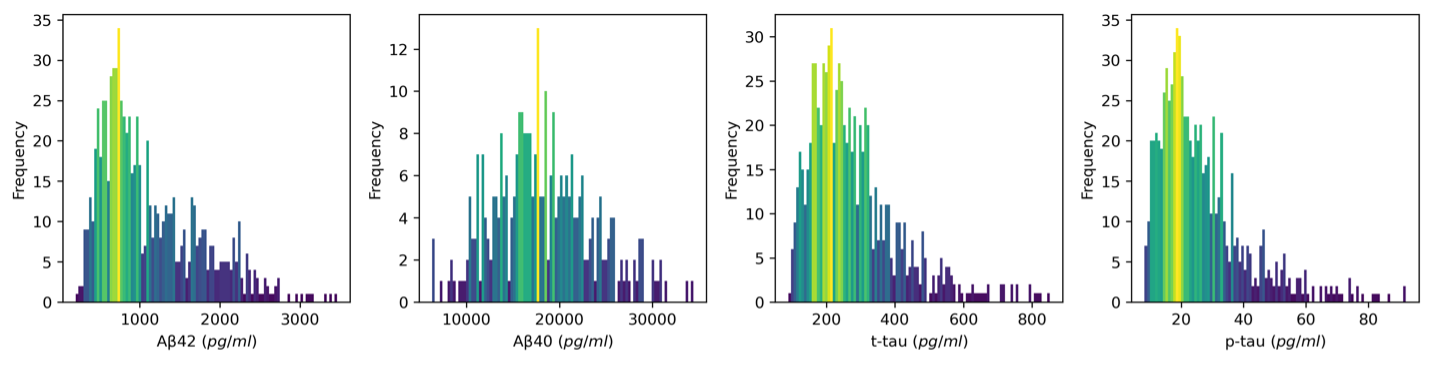


Figure S1. Histogram of proteins level for Aβ42, Aβ40, t-tau, and p-tau.

Figure S2. The fitted linear relationship between the protein level and ChP volume for each study group. β is the standardized regression coefficient.


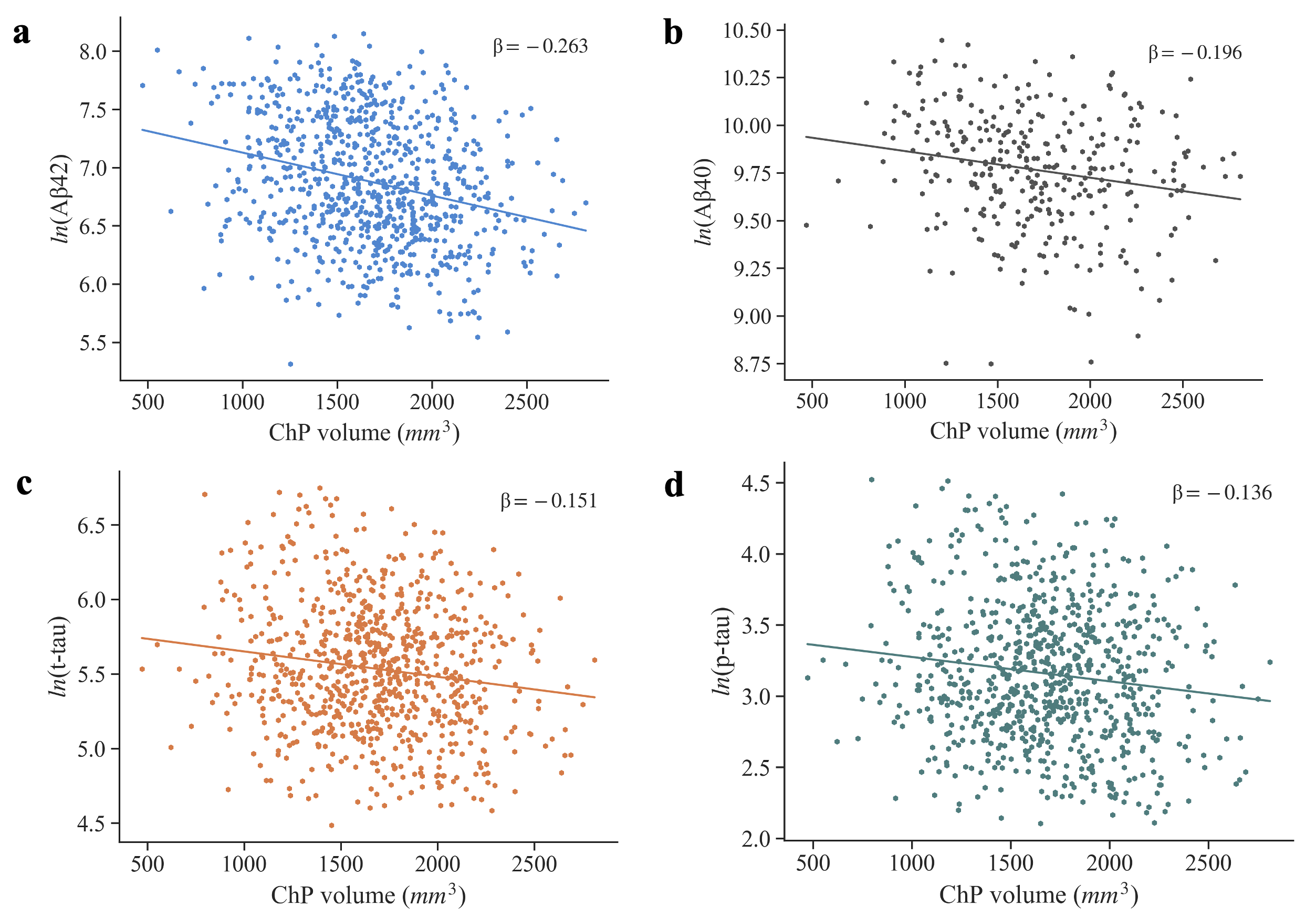


Figure S3. The fitted linear relationship between the protein level and ChP volume. β is the standardized regression coefficient.
